# Infusion Simulation of Graphene-Enhanced Resin in LCM for Thermal and Chemo-Rheological Analysis

**DOI:** 10.3390/ma17040806

**Published:** 2024-02-07

**Authors:** Hatim Alotaibi, Chamil Abeykoon, Constantinos Soutis, Masoud Jabbari

**Affiliations:** 1 Department of Mechanical, Aerospace and Civil Engineering, The University of Manchester, Manchester M13 9PL, UK; 2 Institute of Earth and Space Science, King Abdulaziz City for Science and Technology, Riyadh 12354, Saudi Arabia; 3 Department of Materials, The University of Manchester, Manchester M13 9PL, UK; 4 Aerospace Research Institute, The University of Manchester, Manchester M13 9PL, UK; 5 School of Mechanical Engineering, University of Leeds, Leeds LS2 9JT, UK

**Keywords:** CFD, chemo-rheology, enhancement of thermal properties, graphene, heat transfer, liquid composite moulding

## Abstract

The present numerical study proposes a framework to determine the heat flow parameters—specific heat and thermal conductivity—of resin–graphene nanoplatelets (GNPs) (modified) as well as non-modified resin (with no GNPs). This is performed by evaluating the exothermic reaction which occurs during both the filling and post-filling stages of Liquid Composite Moulding (LCM). The proposed model uses ANSYS Fluent to solve the Stokes–Brinkman (momentum and mass), energy, and chemical species conservation equations to a describe nano-filled resin infusion, chemo-rheological changes, and heat release/transfer simultaneously on a Representative Volume Element (RVE). The transient Volume-of-Fluid (VOF) method is employed to track free-surface propagation (resin–air interface) throughout the computational domain. A User-Defined Function (UDF) is developed together with a User-Defined Scaler (UDS) to incorporate the heat generation (polymerisation), which is added as an extra source term into the energy equation. A separate UDF is used to capture intra-tow (microscopic) flow by adding a source term into the momentum equation. The numerical findings indicate that the incorporation of GNPs can accelerate the curing of the resin system due to the high thermal conductivity of the nanofiller. Furthermore, the model proves its capability in predicting the specific heat and thermal conductivity of the modified and non-modified resin systems utilising the computed heat of reaction data. The analysis shows an increase of ∼15% in the specific heat and thermal conductivity due to different mould temperatures applied (110–170 °C). This, furthermore, stresses the fact that the addition of GNPs (0.2 wt.%) improves the resin-specific heat by 3.68% and thermal conductivity by 58% in comparison to the non-modified thermoset resin. The numerical findings show a satisfactory agreement with and in the range of experimental data available in the literature.

## 1. Introduction

### 1.1. Background

Thermoset resins (e.g., epoxies and polyesters) are increasingly used in aerospace and automotive applications for their suitability in various lamination techniques and the possibility of curing them at room temperature, enhancing mechanical properties, and improving the thermal stability of a structural material. Liquid Composite Moulding (LCM) processes such as Resin Transfer Moulding (RTM), and Vacuum-Assisted Resin Transfer Moulding (VARTM), use thermosets to impregnate a dry fibrous reinforcement to produce composite parts—Fibre-Reinforced Polymer (FRP) composites. In most liquid composite moulding processes, time, temperature, pressure, and flow rates are the processing parameters that affect resin impregnation (infiltration) and polymerisation (curing) during the manufacturing of composites [1,2,3]. The effect of these parameters varies from the formation of voids originating from the fibres’ resistance to flows (permeability) to the degradation of a material due to elevated temperatures. The polymerisation—cross-linking/chemical reaction—of thermosets is an exothermic process that generates heat released by the liquid resin during the filling and curing stages of a liquid composite moulding process cycle. The formation of cross-linked polymer chains (three-dimensional network) affects the resin system by decreasing the polymer molecules’ mobility, and therefore leading to a rapid increase in the resin’s viscosity [1,4,5,6,7]. This is apart from the inclusion (addition) of nanofillers (e.g., graphene) in thermosets which could serve as a means to improve the cross-linking process (catalyst-like effects during preparation/manufacturing), and to enhance the mechanical, physical and chemical properties of the final product. With that being said, the addition could also bring challenges related to morphology (issues relevant to polymer structure at the nano- and macro-scale), and agglomeration (issues relevant to dispersion or nanoparticles size) [8,9]. Such an influence on resin impregnation and polymerisation requires optimised control to acquire industrial processing windows for composites’ manufacturing and characterisation. This is typically carried out using advanced physics modelling tools [4,10,11,12,13].

### 1.2. Reviews and Significance of the Present Work

The modelling and characterisation of cure kinetics (e.g., degree of cure, rate of reaction) and the chemo-rheology (viscosity behaviour) of thermosets has been considerably reported on in the literature with different methodologies [4,12,14,15,16,17,18]. Despite this, a numerical investigation of nanotechnology-based thermosets during LCM processes has not been extensively explored. The nanotechnology-based thermosets involve nano-sized particles known as nanofillers such as widely used Carbon Nanotubes (CNTs), Nanoclays (NCs), and Graphene (G) [19]. Small portions (0.05–0.2 wt.%) of the aforementioned nanomaterials could tailor a resin system through making significant changes to its mechanical, thermal, and physical properties [19]. As such, a graphene layer—bonded carbon atoms in a hexagonal array of ${\mathrm{sp}}^{2}$—has been reported with 5000W/(m·K) thermal conductivity, in addition to unprecedented Young’s modulus and ultimate strength values, i.e., 1TPa, and 130GPa, respectively [20]. Graphene-based materials have impacted a diverse range of industries in recent years including transport, medicine, electronics, energy, and defence [21]. For example, graphene has applications in targeted drug delivery or biosensing for biomedicine, transistors or semiconductors for electronics, and batteries or hydrogen storage for energy [21,22,23,24,25].

This reveals some open research challenges in fabricating graphene using various methods, including the popular mechanical exfoliation method, liquid-phase exfoliation (LPE), and chemical vapour deposition (CVD) [21]. A graphene-enhanced polymer composite is commonly prepared by means of a magnetic stirrer, an ultrasonic homogeniser, and a vacuum heat oven (e.g., de-gasification) [21]. The presence of graphene nanoparticles within a resin system increases its thermal properties, for instance providing specific heat and thermal conductivity, which could impact the curing mechanisms and manufacturing time [26,27,28,29,30]. The measurement of such properties is usually determined using a thermal analysis technique—Differential Scanning Calorimetry (DSC)—or the so-called Laser Flash Analyser (LFA). These experimental measurements are obtainable on a fully cured thermoset sample as this becomes complicated to calculate during curing [31]. However, a relatively new method, Modulated Temperature DSC (MTDSC), has been shown to produce feasible practical measurements of thermal properties during curing, described via the coupling modelisation of heat transfer with kinetics of polymerisation [32,33,34,35].

Umer et al. [36] conducted an experiment to investigate the incorporation of Graphene Oxide (GO) nanoparticles into epoxy resin in different contents. Their study focuses on cure kinetics and the rheology of the modified epoxy resin, in addition to permeability characterisation using vacuum-assisted resin transfer moulding. They concluded that an early cure is observed with the highest content of GO (0.2 wt.%), and hence slowing down flow-front progression (due to viscosity evolution) compared to lower GO contents. A numerical analysis by Nguyen et al. [37] was performed for the flow of graphene-based resin through a porous medium. They used Fluent and MATLAB (Runge-Kutta method) to simulate the filling process (resin front advancement) and to calculate the degree of cure. The temperature- and degree-of-cure-dependent viscosity model was not considered, and instead, the experimentally measured viscosities with or without G, were input—constant values—respectively. Tan et al. [4] integrated the Flux-corrected Transport (FCT) with Finite Element/Control Volume (FE/CV) using PORE-FLOW to study the effect of changing/altering the liquid thermal conductivity on temperature and cure distributions via a representative volume element of a dual-scale fibre preform. Developing a robust numerical model to characterise physical properties is critical for engineering the design of the mould as well as the position of inlets. Hence, the numerical results of Tan et al. [4] showed that an increase in thermal conductivity can impact the convection-dominated flows by increasing the distribution of heat. Since the resin cure is affected by the heat flow, this impact can contribute directly to the curing rate. This, therefore, stresses that tailoring thermal properties via the coupled modelling of filling, curing, and chemo-rheology during composite manufacturing processes would be an optimal solution.

Rafiee et al. [38] characterised the thermal conductivity of a modified epoxy resin with the addition of graphene-based nanofillers (GNPs 1%, GO 2% and reduced Graphene Oxide (rGO) 0.042%) during a vacuum-assisted resin-transfer moulding process. They found an improvement of ∼13.5%—0.383W/(m·K)—in thermal conductivity at 1% GNPs, while that marked relatively lower values of ∼7.75%— (0.368W/(m·K))—and ∼4.87%—(0.358W/(m·K))—for rGO and GO, respectively. Their results also highlighted that a good dispersion of graphene-based nanomaterials together with better control over the interfacial interaction between thermosets and nanofillers are essential for enhancing thermal properties of the manufactured composites. Djebara et al. [39] proposed a modelling approach using FLOW3D to simulate resin infiltration in fibrous media at the mesoscopic scale on a representative volume element. They predicted the effect of different types of nanoparticles on the thermal conductivity of a carbon fibre-epoxy composite. In their simulations, a particle-filled epoxy resin (a loaded flow with particles) was transporting through a dual-scale non-woven fabric for a complete saturation, and a computation of thermal conductivity was performed. Their analysis indicated that the influence of a particle’s thermal conductivity on carbon fibre–epoxy composite was significant, e.g., 42.5W/(m·K) with copper and 9.2W/(m·K) for lead nanomaterials. It is noteworthy that, in their developed numerical approach, curing kinetics and rheological behaviours were not accounted for. A determination of a specific heat during an isothermal cure of a thermosetting polymer was experimentally—using DSC—examined by McHugh et al. [35]. The authors [35] showed that the rate of cure and temperature has a substantial effect on the specific heat which was correlated to an increase in the formation of a cross-link network—around 0.4–0.6 degree of cure. A variation in the specific heat throughout curing was reported, roughly 17–24%, such as 1.9 to 2.23J/(g·K) at 200 °C. McHugh et al. [35] underlined that the specific heat in the vitrification (solid) state, when the majority of the potential cross-links are formed, became merely a function of temperature, and hence independent of cure.

This review of the literature shows that, for any arbitrary (thermoset) resin system and nanoparticles, an analysis like DSC and/or MTDSC is required for a better understanding of the thermal properties—and controlling the manufacturing process [32,33,34,35]. Consequently, the lack of research relating to numerical predictions of heat transfer properties (e.g., specific heat, and thermal conductivity) subject to cure kinetics and chemo-rheological effects is evident. This is crucial for the impregnation of nanotechnology-based thermosets with fibre preforms during liquid composite moulding. Thus, the present work proposes a numerical framework to predict and monitor the specific heat and thermal conductivity of any thermoset resin with the addition of nanoparticles coupled with flow-front, degree of cure, rate of cure, and viscosity evolution models. The methodology adopts the Volume-of-Fluid (VOF) method in ANSYS Fluent based on Finite Volume Method (FVM) discretisation scheme. It furthermore employs Stokes–Brinkman (Equation (3)), energy (Equation (7)), and species (Equation (12)) with supplemental source terms accounting for heat generation (polymerisation) and permeability. User-Defined Functions (UDFs) are created along with User-Defined Scalers (UDSs) to enhance the standard code of ANSYS Fluent for fully coupled thermal, curing, and chemo-rheological models using “DEFINE” macros. This coupled heat-transfer/polymerisation model will be able to simulate and characterise the thermal and chemo-rheological behaviour of graphene(nanofiller)-based thermosets during any LCM process cycle—including the filling and curing (post filling) stages.

## 2. Numerical Model

**Figure 1:**
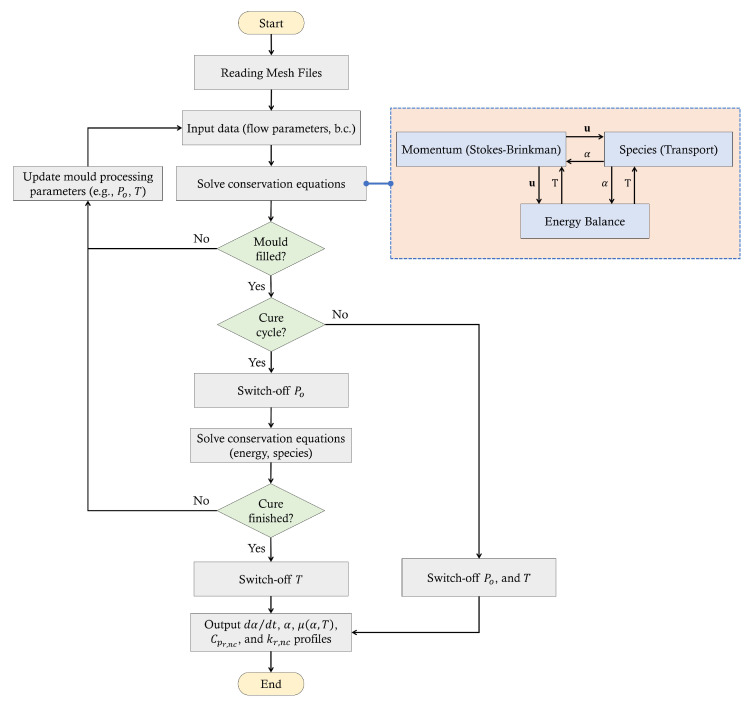
The proposed numerical framework for filling and post-filling (curing) simulation of a LCM process.

The numerical framework solves conservation equations utilising ANSYS Fluent for momentum, continuity, energy, and chemical reactions. The convection–diffusion–reactive flows are simulated adopting the VOF method based on a FVM scheme for discretising the conservation form of the Partial Differential Equations (PDEs). The numerical solver allows for compiling User-defined functions or scalers—C-based codes—that are used to hook the developed functions (e.g., source terms, fluid properties, etc.) to ANSYS Fluent. The reason for this is that the standard features of the commercial code (Fluent) do not supply time/temperature/cure-dependent models (e.g., the chemo-rheology model) which require the customisation of such model parameters by creating UDFs and a defined scalar (i.e., degree of cure) to attain a twinning (linkage) technique for sophisticated thermo-chemo-flow modelling. A flow chart illustrating the developed numerical framework is given in Figure 1.

### 2.1. Momentum and Continuity

The Newtonian Navier–Stokes (N–S) Equation (1) is employed along with the continuity (mass) Equation (2) to solve creeping (viscous) incompressible flow regimes in porous media, as follows

(1) $$\frac{\partial }{\partial t}\left(\rho u\right)+\nabla \;\cdot \;\left(\rho uu\right)=-\nabla p+\mu \nabla^{2}u+\rho g+f$$

(2) ∇·u=0

where u is the volume-averaged velocity, ∇p is the pressure gradient, ρg is the body force term, $\mu \nabla^{2}u$ is the diffusion term, and ∇·(ρuu) is non-linear convective acceleration term. Here, f is a model-dependent source term (i.e., porous model) defined as a resistive force (a viscous resistance) on the flow progression caused by fibres.

The resistance of filaments’ (fibres) flow to advance (resin impregnation) induces a low velocity (i.e., Reynolds number Re≪1), and hence the non-linear convective acceleration term can be neglected. This reduces N–S to the so-called Stokes equations, thereby allowing the simulation of flows within open regions, an inter-tow porosity. The equation of motion incorporates a source term accounting for flow within porous regions, an intra-tow porosity, to enable the dual-scale modelling of the resin impregnation of fibre preforms under variable conditions. This is carried out by applying empirical micro-permeability models (e.g., Gebart [40]) for local-tow impregnation. Such a combination will lead to the well-known Stokes–Brinkman formulation—see Equation (3). The analytical model [40] describes local permeabilities in parallel and transverse to the fibre direction for distinct stacking arrangements. This study considered a hexagonal packing assuming a cylindrical shape of filaments with a 10.5 µm diameter size.

(3) $$\frac{\partial }{\partial t}\left(\rho u\right)-\mu \nabla^{2}u+\nabla p=f$$

This **f** can be equated (e.g., in warp direction) as follows

(4) $$f=\mu K_{t}^{-1}u=\mu \left[\begin{array}{ccc} \frac{1}{K_{t_{∥}}} & 0 & 0\\ 0 & \frac{1}{K_{t_{⊥}}} & 0\\ 0 & 0 & \frac{1}{K_{t_{⊥}}} \end{array}\right]\;\cdot \;\left[\begin{array}{c} u_{\mathrm{xx}}\\ u_{\mathrm{yy}}\\ u_{\mathrm{zz}} \end{array}\right]$$

The model-dependent source term (f) is assigned to porous regions (fibre bundles in our case), in which $K_{t}$ stands for microscopic (intra-tow) permeability, while u and μ indicate the volume-averaged velocity and the time-temperature-cure dependent viscosity respectively. The $K_{t_{⊥}}$ and $K_{t_{∥}}$ are permeations of flow perpendicular (transverse) and along (longitudinal) to fibres. $u_{\mathrm{xx}}$ here is the resin flow velocity in x-direction—this applies to $u_{\mathrm{yy}}$ and $u_{\mathrm{zz}}$ accordingly. Due to the fact that the liquid resin viscosity would exhibit variations over an LCM process influenced by heat transfer and cross-linking reactions, the Castro–Macosko model [41] is employed—a time/temperature/cure-dependent viscosity—which is presented bellow

(5) $$\mu \left(\alpha ,T\right)=\mu_{0}\exp\left(\frac{E_{\mu }}{RT}\right){\left(\frac{\alpha_{\mathrm{gel}}}{\alpha_{\mathrm{gel}}-\alpha }\right)}^{a+b\alpha }$$

where α is the degree of cure (conversion), $\alpha_{\mathrm{gel}}$ is the degree of cure at gelation point, and $E_{\mu }$, *T*, *R* are activation energy in chemo-rheology, temperature, and gas constant, respectively. The other involved parameters like $\mu_{0}$, *a*, and *b* are known as a pre-exponential factors and exponents. The expression of viscosity in Equation (5) is assigned to the ANSYS-CFD solver using a “DEFINE_PROPERTY” macro.

### 2.2. Energy Balance

Heat transfer in the impregnation of resin with fibrous materials occurs during composite manufacturing processes. The general consideration of the energy conservation (e.g., heat transfer) in an RTM process can be explained as follows [42,43]:

(6) $$\begin{array}{c} \left[\phi \rho_{i}C_{p_{i}}+\left(1-\phi \right)\rho_{f}C_{p_{f}}\right]\frac{\partial T}{\partial t}+\rho_{i}C_{p_{i}}u\;\cdot \;\nabla T=-\nabla \;\cdot \;q\\ +\phi \rho_{r}\Delta H\dot{\alpha }\left(\alpha ,T,t\right)+\mu u\;\cdot \;K_{o}^{-1}\;\cdot \;u+\phi \rho_{i}C_{p_{i}}\nabla \;\cdot \;\left(D\;\cdot \;\nabla T\right) \end{array}$$

where $\rho \;[\mathrm{kg}/m^{3}]$, $C_{p}\;\left[J/\mathrm{kg}\;\cdot \;K\right]$, $q\;[W/m^{2}]$, $K_{o}\;\left[m^{2}\right]$, $D\;[m^{2}/s]$, and ϕ[−] are the density, specific heat, heat flux, permeability tensor (global), dispersion tensor, and porosity, respectively. The fibre, resin, and nanocomposite subscripts are defined by f, r, and nc, respectively. ΔH[J/g] is the reaction heat, and $\dot{\alpha }\left(\alpha ,T,t\right)$ stands for the rate of cure reaction.

The first and second terms on the left-hand side of Equation (6) denote unsteadiness and convection, while fisrt, second, third, and fourth terms on the right-hand side indicate diffusion, reaction, viscous dissipation, and thermal dispersion, respectively. Using Fourier’s law for the heat flux ($q=-\left(K_{T}\;\cdot \;\nabla T\right)$) leads to the conduction term ($\nabla \;\cdot \;\left(K_{T}\;\cdot \;\nabla T\right)$) in Equation (7).

The convection phenomenon between fibres and resins can be neglected in creeping flows owing to the small Graetz number (Gz≪1)—the ratio of heat transfer by convection (in-plane) to that by conduction (through-thickness) [42,43]. Thus, a thermal equilibrium approach can be followed, wherein porous media and impregnating fluids share the same temperature at each point [14,15,44,45]. The so-called dimensionless groups such as Brinkman number (Br), and Péclet number (Pe)—see [42,43] for details—can describe the importance of viscous dissipation and thermal dispersion, respectively. In our case, Br and Pe are very low (approaching zero), thus, viscous dissipation and thermal dispersion terms are of marginal importance, and can be eliminated. This will reduce the above-mentioned energy balance Equation (6) to Equation (7) as shown below [42,43,46,47,48]: Thence, the heat transfer (energy balance for incompressible flows) during the LCM process becomes [42,43,46,47,48]:

(7) $$\rho C_{p}\frac{\partial T}{\partial t}+\rho_{i}C_{p_{i}}\left(u\;\cdot \;\nabla T\right)=\nabla \left(K_{T}\;\cdot \;\nabla T\right)+\phi \rho_{i}\Delta H\dot{\alpha }\left(\alpha ,T,t\right)$$

(8) $$\left\{\begin{array}{c} \rho =\frac{\left(\rho_{f}\rho_{i}\right)}{\left(\rho_{f}w_{i}+\rho_{i}w_{f}\right)}\\ K_{T_{\|}}=w_{i}k_{i}+w_{f}k_{f},\;K_{T_{⊥}}=\frac{\left(k_{f}k_{i}\right)}{\left(k_{f}w_{i}+k_{i}w_{f}\right)}\\ C_{p}=w_{i}C_{p_{i}}+w_{f}C_{p_{f}}\\ w_{i}=\frac{\left(\phi /\rho_{f}\right)}{\left(\frac{\phi }{\rho_{f}}+\frac{1-\phi }{\rho_{i}}\right)}\\ w_{f}=1-w_{i}\\ i=\mathrm{resin}\;\left(r\right)\;\mathrm{or}\;\mathrm{nanocomposites}\;\left(\mathrm{nc}\right) \end{array}\right.$$

(9) $$\left\{\begin{array}{c} \rho_{\mathrm{nc}}=\frac{\rho_{r}\rho_{\mathrm{filler}\;}}{\psi_{\mathrm{filler}\;}\rho_{r}+\left(1-\psi_{\mathrm{filler}\;}\right)\rho_{\mathrm{filler}\;}}\\ k_{\mathrm{nc}}=k_{r}\left[\frac{k_{\mathrm{filler}\;}+2k_{r}+2\psi_{\mathrm{filler}}\left(k_{\mathrm{filler}\;}-k_{r}\right)}{k_{\mathrm{filler}\;}+2k_{r}-\psi_{\mathrm{filler}\;}\left(k_{\mathrm{filler}\;}-k_{r}\right)}\right]\\ C_{p_{\mathrm{nc}}}=\left(1-\psi_{\mathrm{filler}\;}\right)C_{r}+\psi_{\mathrm{filler}\;}C_{\mathrm{filler}\;} \end{array}\right.$$

where $w\;\left[-\right]$, $\psi_{\mathrm{filler}}\;\left[-\right]$, and $K_{T}\left[W/m\;\cdot \;K\right]$ are weight fraction, filler volume fraction, and the thermal conductivity tensor, respectively. The thermal conductivity tensor $\left(K_{T}\right)$ is denoted by diagonals $K_{T_{\|}}$ (in-plane) and $K_{T_{⊥}}$ (out-plane), and due to the symmetry in such an orthotropic structure, the off-diagonal terms are set to zero [12,47]. The liquid resin’s viscosity is heavily dependent on temperature, whereby an initiation of cure starts to take a place. In such a manner, this will generate heat as a result of exothermic reactions. The specific heat can be represented by the enthalpy change: a heat transfer in a material system due to chemical reactions (see Equation (10)), through which the quantity of heat required to raise the temperature of the polymer system is measured [33,35,49]. This includes the heat flux—total heat flow rate, $Q_{\mathrm{tot}}$, and residual heat flow, $Q\left(t,T\right)$—of a thermoset polymer with weight *m* attributable to exothermic reactions under a heating rate [33,35,49].

(10) $$C_{p_{i}}=\left\{\begin{array}{cc} \frac{\partial H_{r}}{\partial T} & ,\;wt.\%\mathrm{nanofiller}\;=0\\ \frac{\partial H_{\mathrm{nc}}}{\partial T} & ,\;wt.\%\mathrm{nanofiller}\;>0\\ \; & \frac{\partial H_{i}}{\partial T}=\frac{\frac{dQ_{\mathrm{tot}}}{t}-Q\left(t,T\right)}{\frac{dT}{t}}\;\cdot \;\frac{1}{m} \end{array}\right.$$

Considering slow flow conditions, which are common in RTM/VARTM, the Péclet number is found to be small (approaching zero), wherefore the thermal diffusivity would show negligible variations in the temperature range and can be assumed constant [46,48]. On that account, the thermal conductivity for flowing and curing liquids can be determined using the relationship within the formula for heat transfer rate, as follows

(11) $$k_{i}=\left\{\begin{array}{cc} C_{p_{r}}\rho_{r}D_{r} & ,\;wt.\%\mathrm{nanofiller}=0\\ C_{p_{\mathrm{nc}}}\rho_{\mathrm{nc}}D_{\mathrm{nc}} & ,\;wt.\%\mathrm{nanofiller}>0 \end{array}\right.$$

where $k_{i}$ is the thermal conductivity of resin (r) or nanocomposites (nc), and *D* is the thermal diffusivity.

The specific heat and thermal conductivity expression are all written into UDFs coupled with a UDS to model heat transfer parameters during liquid moulding of composites. This numerical method applies to graphene-enhanced (nanotechnology-based) and non-modified thermoset resins. Material properties, namely, density and thermal diffusivity of the modified thermoset resin, are calculated by the rule of mixture [50]. The developed UDFs use “DEFINE_PROPERTY” macros to integrate heat transfer properties, i.e., specific heat and thermal conductivity of the thermoset polymer, into the set of conservation equations. Whereas, the heat generation term is given by a UDF defined by a “DEFINE_SOURCE” macro.

### 2.3. Species Transport

In the event of convective flows, the conservation of species (caused by chemical reaction) for the liquid resin is expressed by the continuity equation (mass transfer equation)—Equation (12). This describes the convective-dominated transport phenomena by the velocity field of the fluid transporting species, in particular, the degree of cure. The first and second terms at the left-hand side denote unsteadiness (transient) and convection, respectively. The reaction rate of the resin is added as a source term—the right-hand side—to attain an expression that characterises the conversion of monomers to polymers. A scaler quantity, the degree of cure, is considered as a field variable that is associated with its relevant phase domain with which the corresponding supplied UDFs are computed.

(12) $$\phi \frac{\partial \alpha }{\partial t}+\left(u\;\cdot \;\nabla \alpha \right)=\phi \dot{\alpha }\left(\alpha ,T,t\right)$$

The reaction rate, $\dot{\alpha }\left(\alpha ,T,t\right)$, in the energy balance (Equation (7)) and mass transfer Equation (12) requires details of kinetic parameters to describe polymerisation rate, and to contribute to the heat generation term. The kinetic expression follows the so-called Kamal (or modified Kamal) model—an autocatalytic-type—as given below [51,52,53,54]:

(13) $$\dot{\alpha }\left(\alpha ,T,t\right)=A_{0}\exp\left(\frac{-E_{a}}{RT}\right)\alpha^{m}{\left(1-\alpha \right)}^{n}$$

where $\dot{\alpha }\left(\alpha ,T,t\right)$ is the rate of the reaction. The exothermic activation energy is denoted by $E_{a}$, and the universal gas constant is *R*, while $A_{0}$ is a pre-exponential factor. Factors *m* and *n* indicate exponents.

### 2.4. Boundary Conditions and Geometry Details

**Figure 2:**
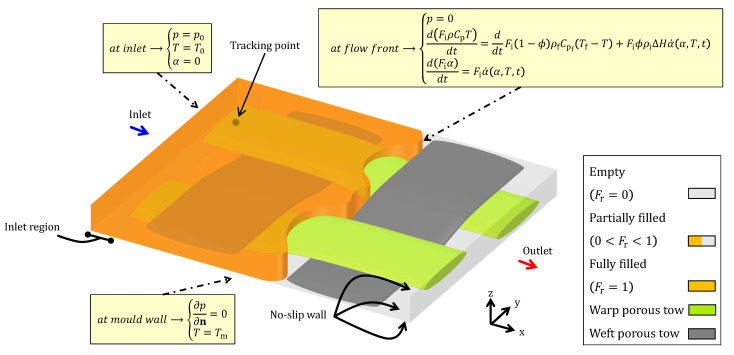
Boundary conditions (b.c.) and RVE geometry used in the simulations.

The thermo-chemo-flow simulations are performed on a Representative Volume Element (RVE) model. This RVE of a woven fabric, a plain weave, is designed by ANSYS DesignModeler—a feature-based parametric solid and surface CAD (Computer-Aided Design) modeler. This is followed by meshing, whereupon a mesh-independence study is carried out to acquire appropriate and accurate solutions. For further details, the current authors [55,56] conducted and thoroughly discussed a convergence and grid (mesh) independence study in their previous numerical works. Such an RVE will include open (inter-tow) and porous (intra-tow) regions to quantify a two-scale (micro-meso) fill model that will yield a value representing the macro-scale level of the resin flow. Figure 2 illustrates the boundary conditions (b.c.) within a rectangular RVE domain 6.56mm×5.66mm×0.575mm comprising an extended inlet region of 0.5mm. The inputs required for the thermo-chemo-flow model, for instance material and process parameters, and simulation/discretisation details/schemes, are summarised in Table 1 and Table 2, respectively. The experimental data (of García-Martínez et al. [27]) adopted in the present work does not supply exponents values, and hence, they are assumed as 1.5 and 1 for *a* and *b* based on the work by Shojaei et al. [13].

## 3. Results

The filling simulation is performed at an injection pressure of 50kPa and a bottom-wall temperature of $110{\;}^{\circ }C$. It is worth mentioning that setting the infusion temperatures is contingent on a polymeric matrix type, for this reason, the above-mentioned temperature is the recommended processing (fill stage) temperature [27]. The User-Defined Functions (UDFs) applied at the boundary conditions, the pressure injection, and processing temperature monitor and control the filling and post-filling (cure) stages. This includes switching between different injection pressures and mould temperatures to stop resin infusion (after complete filling) and increase the mould-wall temperature, whereby the processing mould temperature is maintained at $110{\;}^{\circ }C$ during the filling process for each numerical simulation. While this was kept the same over the post-filling period of time for the first case, it nonetheless increased within 60s to $130{\;}^{\circ }C$, $150{\;}^{\circ }C$, and $170{\;}^{\circ }C$ for the other numerical cases, respectively. Section 3.1 and Section 3.2 investigate the presence of GNPs in thermosets curing reaction and chemo-rheology, and demonstrate numerical predictions of the heat transfer parameters—specific heat and thermal conductivity.

### 3.1. Cure Kinetics and Chemo-Rheology Modelling

**Figure 3:**
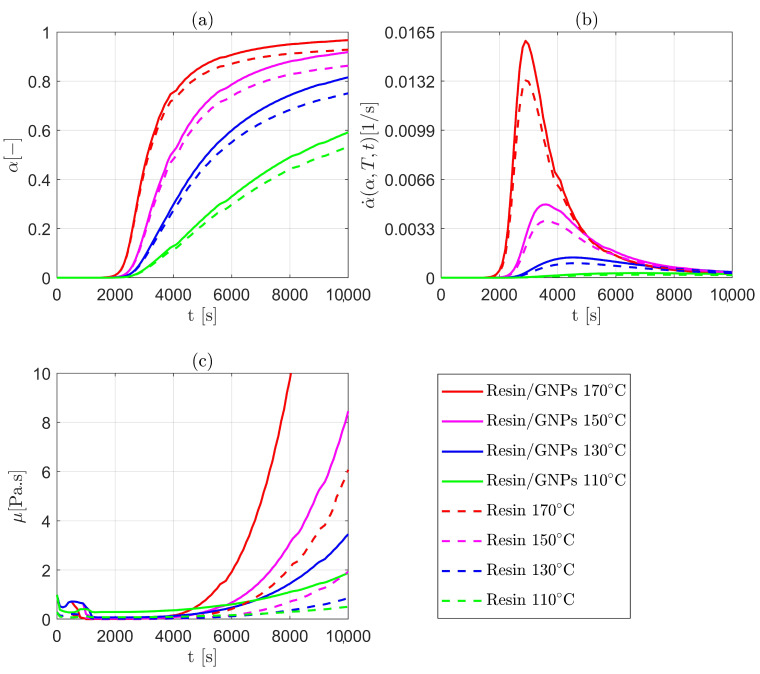
Numerical results for the cure and chemo-rheology of resin/GNPs (modified) and resin (non-modified) during LCM process: (**a**) degree of cure (chemical conversion); (**b**) rate of crosslinking reactions; and (**c**) development of viscosity (rheology).

The effect of nanofillers, i.e., graphene nanoplatelets (GNPs), on cure kinetics and chemo-rheological behaviour is investigated. In general, the polymerisation (cross-linking process) advances during the pre-cure (filling) and cure stages, determining the material state of such graphene reinforced polymer nanocomposites. With heating and time, the modified (nano-filled) liquid resin starts to change from being short monomers (liquid) to becoming cross-linked networks of the formed polymer chains (glassy). From Figure 3a,b, the influence of graphene nanoplatelets can be observed in the context of accelerating the conversion $\left(\alpha \right)$, and consequently the rate of reaction $\left(\dot{\alpha }\left(\alpha ,T,t\right)\right)$. Moreover, the curing rate—c.f., Figure 3b—appears to be higher (e.g., 0.0156 $s^{-1}$ vs. $0.0131\;s^{-1}$ at $170{\;}^{\circ }C$) for the modified resin system compared to the non-modified one. Such disparities are ascribed to the GNPs functionalisation of the resin system. The results can also be variable by virtue of thermoset or nanofiller types as well as the nanofiller content by weight (wt.%). The UDFs created utilise User-Defined Memory (UDM) macros to store field values, i.e., rate of reaction values that are derived from computed values of the coupled UDF-UDS. Figure 3c shows numerical chemo-rheology results when adopting the Castro–Macosko model. As can be seen, the viscosity initially drops because of the rise in temperature, and this subsequently becomes vital upon the formation of cross-linked molecular networks. Accordingly, the viscosity will dramatically increase which can affect the impregnation of the resin into fabrics and its processing time. The evolution of the convection–diffusion–reactive flows in open regions (areas in between yarns) and porous regions (areas within yarns) are found to be roughly identical owing to the assumption of the thermal equilibrium between the fibre and the resin flow.

### 3.2. Specific Heat and Thermal Conductivity Modelling

Numerical determination of specific heat for modified and non-modified thermoset resins follows the general theory—see Equation (10)—used in experimental DSC or the so-called Modulated Temperature DSC (MTDSC) measurements. With this expression, the developed UDF that is specifically designated for the heat of liquids exploits the calculated heat fluxes via UDS; in other words, the data for the total and residual heat of the reaction within the resin/GNPs system are computed to obtain specific heat values. From the rate of heat transfer (i.e., thermal diffusivity coefficient) through a material, the heat equation promotes the calculation of thermal conductivity as a function of volumetric heat capacity and thermal diffusivity—see Equation (11). Hereby, the thermal conductivity UDF uses a predefined function (specific heat macro) in the code to return or retrieve values for a simultaneous prediction of the thermal conductivity of the liquid resin throughout the moulding process (fill and cure stages).

**Figure 4:**
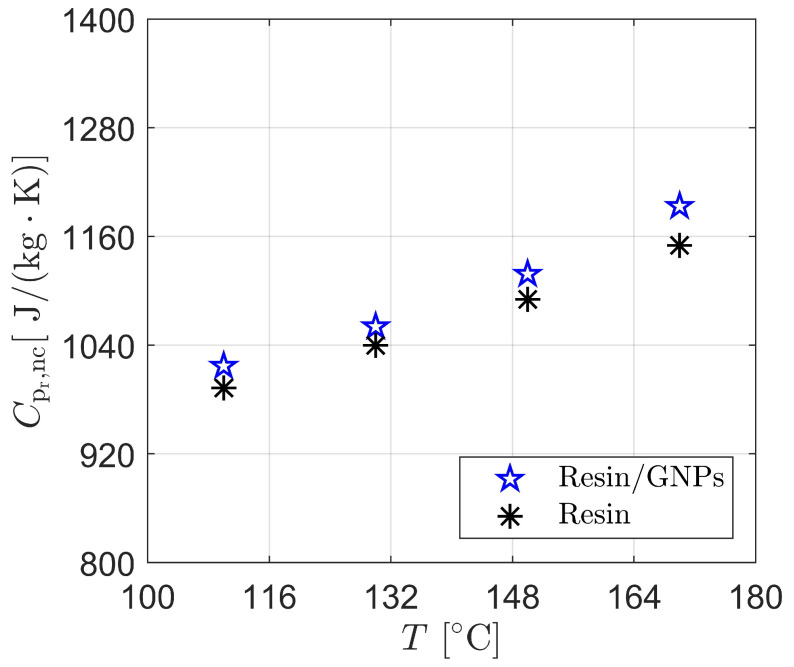
Numerical determination of specific heat in resin/GNPs (0.2 wt.%) and “neat” resin during LCM process: Enhancements in the specific heat (higher) are observed with the additions of GNPs to the thermoset polymer.

Figure 4 depicts numerical estimates of maximum specific heat values of the graphene-based and non-graphene-based thermoset resins during the impregnation and cure of woven fabrics. It is found that an increase in temperature gives rise to the specific heat due to higher cure rates (exothermic reactions) that are driven by higher heating rates, and hence, higher heat flows. An example can be given with $110{\;}^{\circ }C$ and $170{\;}^{\circ }C$ mould temperatures, in which 992J/(g·K) at $110{\;}^{\circ }C$ and 1016J/(g·K) at $110{\;}^{\circ }C$ are achieved for modified and non-modified resin flows, respectively, which increased (at $170{\;}^{\circ }C$) by 14–16% reaching 1150J/(g·K) and 1193J/(g·K) for each. The effect of GNPs on resin thermodynamic properties is likewise attributed to the total heat reaction and cure rate, thereby resulting in an increase ranging from 1.9% to 3.68% (e.g., 1150J/(g·K) to 1193J/(g·K) for resin/GNPs).

**Figure 5:**
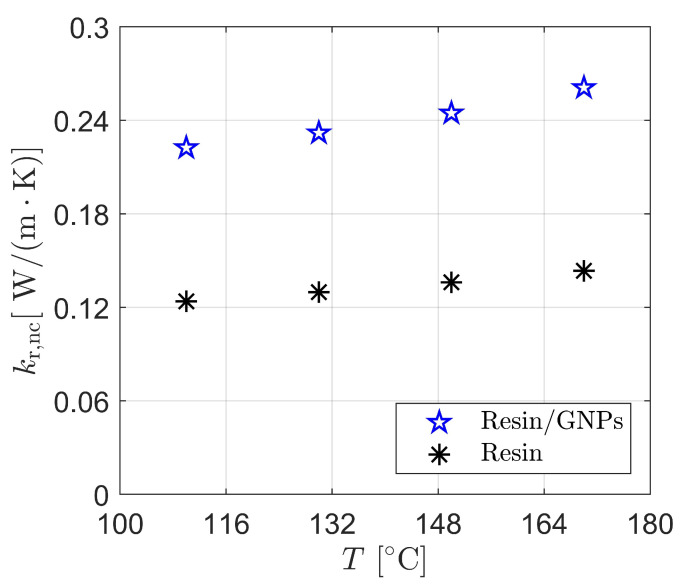
Numerical calculation of thermal conductivity in resin/GNPs (0.2 wt.%) and “neat” resin during LCM process: GNPs serves as a property (thermal conductivity) enhancer and hence a cross-linker.

Similarly, the thermal conductivity of the fluid flow shows similar trends, wherein the difference, with respect to temperature, is approximately ∼15%—c.f., Figure 5. This discrepancy becomes pronounced (∼56–58%) at the additional weight contents (wt.%) of graphene; for instance, the computed thermal conductivities indicate 0.149W/(m·K) and 0.26W/(m·K) at $170{\;}^{\circ }C$ for 0 wt.% and 0.2 wt.% accordingly. Such an enhancement can be explained by the volumetric heat capacity, particularly the density and specific heat of the nanoparticle-filled polymers (mixture). Figure 5 also reveals that more additions (more GNPs %wt.) could contribute to the enhancement of thermal conductivity; however, this might not be always the case as agglomerations may possibly occur, which could consequently alter the physical and mechanical integrity of the manufactured part. Preparation methods (e.g., in-situ polymerization) and graphene types (e.g., graphene oxide (GO), graphene nanoplatelets (GNPs), etc.) can furthermore influence thermal conductivity in a different manner—check [28,57] for further details. García-Martínez et al. [27,58] measured the thermal conductivities of such systems (see Table 1 and Table 3) using TPS 2500S—a thermal analyser instrument—on a fully cured samples that underwent heating to $110{\;}^{\circ }C$ with an increase to ∼$185{\;}^{\circ }C$ in processing temperatures during the cure cycle. In a similar manner, the thermal conductivities predicted by the developed numerical framework (with $110{\;}^{\circ }C$, and $170{\;}^{\circ }C$ bottom-wall temperatures for the filling (semi-cure) and curing stages, respectively) show a satisfactory agreement (e.g., 20.7%) with [27,58]—see Table 3. The discrepancies can be attributed to the autocatalytic-type reaction (see Equation (13)) employed by the present work. This is due to the fact that such an autocatalytic model neglects the diffusion-controlled mechanism that occurred post-vitrification. While not the only errots to occur, experimental uncertainties, such as those in [58], are more likely to arise during the measurement process, hence this might be the reason for the discrepancies with [58]. It is worth mentioning that the experiment in [58] did not provide measurements for thermal diffusivities, thereby, such data were obtained from the literature [59,60] for similar resin types and GNPs. This assumption of such a critical parameter (affecting Equation (11)) could cause disparities in the validation of the numerical framework. Furthermore, the numerical results stress that the calculated thermal conductivity improvements (∼56–58%) fall within the reported practical measurement range for graphene-enhanced thermosetting resins [28,57,61,62,63,64].

**Figure 6:**
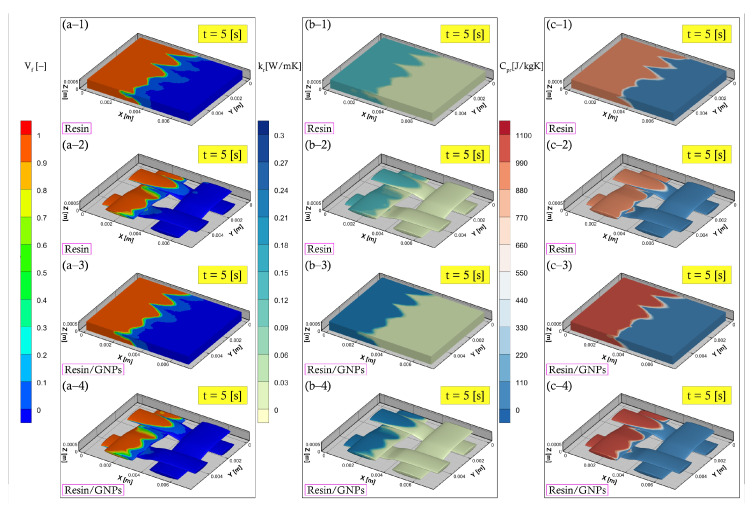
Numerical simulations during filling stage at a bottom-wall temperature of $110{\;}^{\circ }C$: (**a**) resin flow advancement (**a–1**,**a–3**) entire RVE (**a–2**,**a–4**) fibre bundles, (**b**) thermal conductivity mechanisms (**b–1**,**b–2**) no nanofillers (**b–3**,**b–4**) with nanofillers, and (**c**) specific heat development of non-modified resin (**c–1**,**c–2**) and modified resin (**c–3**,**c–4**).

**Figure 7:**
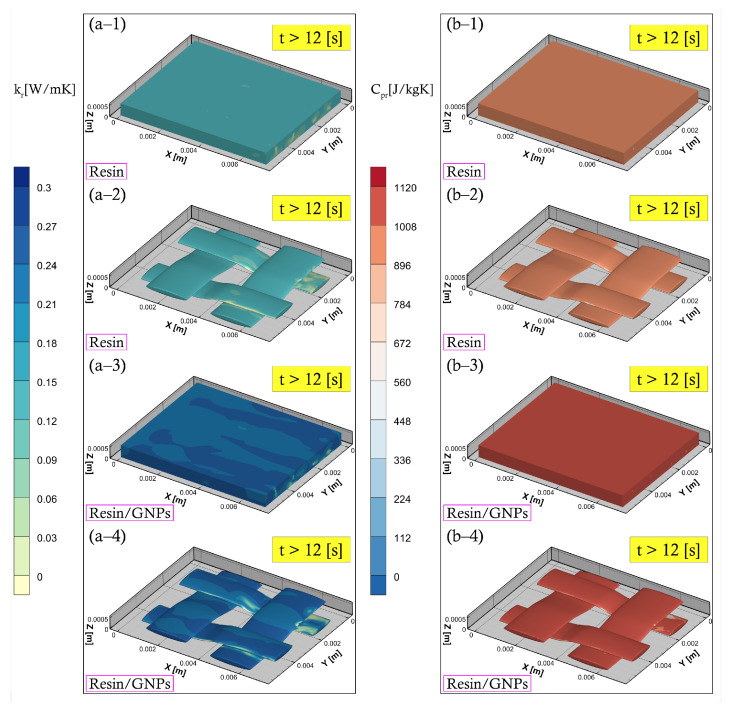
Numerical simulations during post-filling (cure) stage at an increased bottom-wall temperature of $170{\;}^{\circ }C$: (**a**) thermal conductivity distribution in the cure cycle (**a–1**,**a–3**) entire RVE (**a–2**,**a–4**) fibre bundles with higher conductive mechanisms in graphene-enhanced resins—see (**a–3**) or (**a–4**)), and (**b**) specific heat (**b–1**,**b–3**) entire RVE (**b–2**,**b–4**) fibre bundles with increased in Resin/GNPs due to higher exothermic reactions—see (**b–3**) or (**b–4**)).

The development of the heat flow parameters during the filling and post-filling stages are illustrated in Figure 6 and Figure 7, respectively. This is an e-monitoring feature that is efficient in describing liquid phase progression and property behaviour changes utilising the VOF approach. Thermal conductivity and the specific heat of the resin system increase in the early stage of the impregnation process due to the exothermic (cross-linking) reaction triggered by heating temperature—see Figure 6. Such an increase will reach a maximum and then stabilise until bottom-wall temperature disconnection. The nanofillers’ impact can be seen in Figure 6 and Figure 7, accelerating the movement of heat within the liquid resin, stimulating higher rates of reaction, and hence higher (enhanced) heat transfer properties—e.g., comparing (b-2) and (b-4) in Figure 6, or (a-2) and (a-4) in Figure 7.

Coupling flow–energy–species equations using FVM-VOF required stabilisation methods to eliminate numerical diffusion (error/divergence). These methods could include mesh improvement (e.g., converting highly skewed cells to polyhedra), the employment of high-order upwinding/interpolation schemes, and the use of optional relaxation factors. The sophisticated numerical framework in the present work should be able to model complex flows in LCM, which becomes indispensable in the case of incorporating graphene nano-reinforcements into thermosets.

## 4. Conclusions

The incorporation of nanofillers—graphene nanoplatelets—into polymeric matrices is numerically investigated in terms of heat transfer and cure kinetics variables. The present computational study employs Stokes–Brinkman, energy, and species equations to characterise nano-filled resin flow, cure reaction, and chemo-rheology in liquid moulding of woven fabrics. On that premise, the coupled thermo-chemo-flow model uses “DEFINE” macros from ANSYS Fluent to integrate UDF-UDS-based source terms and functions. This allows for the computation of the total and residual heat in a reaction within the thermoset/graphene system, and hence the calculation of specific heat and thermal conductivity.

The results show that the addition of GNPs accelerates the cross-linking reaction by changing the functionality of the exceptional heat transfer properties that graphene possesses. This implies a reduction of ∼3% in the peak temperature, causing an earlier gelation in the fluid flow; as a case in point, a rapid increase in viscosity at $170{\;}^{\circ }C$ initiates after ${6\times 10}^{3}\;s$, whereas this lags behind the ${7.9\times 10}^{3}\;s$ for the non-modified thermoset resins. Numerical determination of specific heat utilises a UDF-UDS-based function that exploits the computed values of the total and residual (response of chemical transformation) heat flows. By this means, the liquid thermal conductivity is concurrently calculated using the formulated relationship—see Equation (11). The numerical framework developed in the present study is an imperative “toolbox”—new technique—that provides information on the heat transfer characteristics during composites manufacturing in an effort to enhance the overall efficiency of the final product. A future work would waive the assumption of thermal equilibrium between fibres (solids) and multiphase fluid flows (i.e., air and resin) to consider a non-equilibrium (thermal imbalance) model for characterising the influence of heat flux within porous media solids on fluid zones.

## Figures and Tables

**Table 1:** Material and processing parameters employed within the numerical study.

Description	Parameter	Unit	Resin System	Resin System + wt. 0.2% GNPs
Resin Moulding of Fabrics [27]	ρ	$\mathrm{kg}/m^{3}$	1260	1271
	*D*	$m^{2}/s$	$0.99\times 10^{-7}$	$3.75\times 10^{-6}$
	$p_{0}$	kPa	50	50
Rheology [13,27]	$\mu_{0}$	Pa·s	$1.1325\times 10^{-14}$	$2.2263\times 10^{-13}$
	$E_{\mu }$	J/mol	94,200	88,900
	R	J/(mol·K)	8.3144	8.3144
	$\alpha_{\mathrm{gel}}$	—	0.1	0.1
	*a*	—	1.5	1.5
	*b*	—	1	1
Cure Kinetics [27]	$A_{0}$	$s^{-1}$	$2.81\times 10^{10}$	$7.67\times 10^{9}$
	$E_{a}$	J/mol	97,540	91,883
	*m*	—	1.2	1.2
	*n*	—	1.7	1.8
	ΔH	J/kg	$441\times 10^{3}$	$452\times 10^{3}$
Fabric Design Parameters [56]:				
$K_{t_{\|}}\;\left[m^{2}\right]=2.08\times 10^{-13}$		$\rho_{f}\;\left[\mathrm{kg}/m^{3}\right]=2536$		
$K_{t_{⊥}}\;\left[m^{2}\right]=2.71\times 10^{-14}$		Width warp yarns [mm]=1.63		
$V_{f}\left[\%\right]=50$		Gap warp yarns [mm]=1.16		
$\phi_{o}\left[\%\right]=50$		Width fill yarns [mm]=2.05		
$\phi_{t}\left[\%\right]=20$		Gap fill yarns [mm]=1.09		
$\phi_{s}\left[\%\right]=37.5$				

**Table 2:** Simulation details and discretisation schemes.

Mesh	Detail	Solution	Method
Mesh elements (N)	∼4 M	Algorithm	SIMPLE
Mesh method	Polyhedra	Convection term	Second-order upwind
Skewness	<0.8	Volume fraction	VOF
Mesh size	Adaptive sizing (ranging from 0.05 to 0.5 mm)	Time-stepping	Implicit
		Stepping size	5 s
SIMPLE: Semi-implicit method for Pressure-linked Equations.			
VOF: Volume of Fluid formulation is a time-dependent solution for multiphase flow problems.			

**Table 3:** Numerical calculations versus experimental measurements for modified and non-modified resin systems.

System	$k_{i}\;\left[W/m\;\cdot \;K\right]$		% Error
	García-Martínez et al. [27,58] (Expt.)	Present Work (Num.)	
Resin	0.207	0.149	38
Resin/GNPs (0.2 wt.%)	0.32	0.265	20.7

## Data Availability

Data are contained within the article.

## Abbreviations

*Latin letters:*
$A_{0}$	Pre-exponential constant [1/s]
$C_{p}$	Specific heat [J/kg·K]
D	Dispersion tensor $\left[m^{2}/s\right]$
*D*	Thermal diffusivity $\left[m^{2}/s\right]$
$E_{i}$	Activation energy [J/mol]
f	Model-dependent source term
g	Gravitational acceleration $\left[m/s^{2}\right]$
*H*	Reaction heat [J/g]
$K_{T}$	Thermal conductivity [W/m·K]
$K_{o}$	Permeability tensor $\left[m^{2}\right]$
$K_{t}$	Intra-tow permeability $\left[m^{2}\right]$
*p*	Pressure [Pa]
q	Heat flux $\left[W/m^{2}\right]$
*R*	Universal gas constant [J/mol·K]
*T*	Temperature [K]
*t*	Time [s]
u	Volume averaged velocity [m/s]
$V_{f}$	Fibre volume fraction [−]
$w_{f,r}$	Weight fraction [−]
*Greek letters:*
$\dot{\alpha }$	Rate of reaction [1/s]
α	Degree of cure [−]
μ	Dynamic viscosity [Pa·s]
ρ	Density $\left[\mathrm{kg}/m^{3}\right]$
ϕ	Porosity of the medium [−]
$\psi_{i}$	Nanofiller fraction [−]
*Subscripts:*
‖	Longitudinal/parallel
⊥	Transverse/perpendicular
f	Fibre/filament
gel	Gelation point
nc	Nanocomposites
o	Overall/global
r	Resin
s	Inter-tow/mesoscopic
t	Intra-tow/microscopic
x,y,z	Global coordinate system
*Superscript:*
a,b,m,n	Exponents
